# A Case of Sudden Death in Ovariotomy, While the Patient Was under the Influence of Chloroform

**Published:** 1870-04

**Authors:** James Y. Simpson


### Miscellaneous.

### A Case of Sudden Death in Ovariotomy, while the Patient was under the Influence of Chloroform.

BY SIR JAMES Y. SIMPSON, M. D.

A few weeks ago, Mr. Brotherston, of Alloa, sent to Edinburg a patient who was the subject of an ovarian tumor. She brought a note from him, asking if I thought the case a fit one for ovariotomy. I wrote back that it seemed to me to be so. The patient was married, about 22 years of age, thin and emaciated; and I thought that the tumor, which was as large as the pregnant uterus at the sixth and seventh month, felt more solid than multilocular ovarian tumors of this size usually do; yet it seems free from adhesions.

Mr. Brotherston took the patient into the small village hospital at Alloa, and earnestly requested me to be present when he operated. Accordingly, on the morning of February 5th, I went up to him. Dr. Wilson and Duncanson, of Alloa, were also to be present; but Dr. Duncanson did not arrive till after the patient was laid upon the table and the operation begun. With the view of allowing Dr. Wilson to give full assistance to Mr. Brotherston at the operation, I chloroformed the patient. In doing so, I placed a single layer of towel over the nose and mouth, leaving the eyes exposed; and dropped the chloroform upon the towel. When Mr. Brotherston made his first continuous incision, the patient moved so much that he stopped for a short time, till I put the patient more under the effects of the anæsthetic. The tumor was rapidly reached, and was attempted to be diminished in size by tapping; but only a comparatively small quantity of fluid escaped. Mr. Brotherston then extended the opening upward for an inch or more above the umbilicus, and was introducing and using his hand with the view of turning out the ovarian mass, when the patient vomited suddenly and profusely. Immediately the eyes opened; the pupils were preternaturally dilated; the face looked pallid; and the respiration which had never been affected by the chloroform so as to have the least noise or stertor in it, seemed arrested. Instantly artificial respiration was set on foot, and the tongue was pulled forward. Deep spontaneous respiration then occurred several times in succession, and I deemed at the moment that the patient was out of danger; but a second collapse occurred, which terminated in death, all means of resuscitation proving unavailing:

On a *post-mortem* examination of the body, ordered by the legal authorities, no disease could be found in the head or chest, or elsewhere. The ovarian tumor was free from any peritoneal adhesions. On examing its structure, Dr. Pettigrew, the esteemed pathologist of the Royal Infirmary of Edinburg, found it to be cancerous in its character.

Remarks: *Cases of Sudden Death during Surgical Operations without Anœsthetics.*—In the first paper which I published on Chloroform in the *Edinburg Monthly Journal of Medical Science* for December, 1847, I stated that this drug, if given in too great or too long doses, "would doubtless produce serious consequences and even death; and at the same time I expressed the hope that "its great potency would be one great safeguard against its abuse." Since that period I have administered it myself, or been present when it was administered, in several thousands of instances; but have not seen its employment terminate in death before the occurrence of the preceding unhappy case.

According to all the experimental and clinical observations which have been made, chloroform appears capable of destroying life in two ways—namely, (1) asphyxia, and (2) by syncope. Death by asphyxia can generally, if not always be averted, by at once arresting the inhalation of the drug whenever the breathing becomes noisy or stertorous—states, which are already mentioned, never occurred with the preceding patient. Syncope, or sudden stoppage of the action of the heart, is doubtless far less under control, and has apparently formed the principal cause of the fatal issue in almost all the cases in which patients have perished when under the use of chloroform. Perhaps fewer cases of syncope actually do occur under operations since the introduction of anæsthetics, because the nervous and sensory system of the patients are so far obtunded by their employment; but a patient is, I believe, in greater jeopardy if syncope do happen while he is under the influence of chloroform, than when he is not under it, because the irritability and action of the heart are diminished by the free use of it, as shown by the lowering and slowing of the pulse. Yet, when syncope does occur in chloroformed persons, artificial respiration and its accompaniments are usually sufficient to rally and restore the patient. When the preceding case was described by me at a late meeting of the Edinburg Obstetrical Society, Dr. Gordon and Dr. Andrus McDonald each mentioned an instance in which sudden fainting took place, with pallor of the face, open eyes, and very dilated pupils at the first commencement of the incision in two slight operations—the one for the removal of a small tumor, the other for the incision of a carbuncle; and in neither case had the patient taken any unusual dose of chloroform. They both recovered under artificial respiration. Several analogous cases have been recorded.

But are all such cases of syncope, which take place during operations, and which end, or do not end in death, the result of the action of the chloroform which happens to be used at the time? The question is one which has never perhaps sufficiently attracted the attention of the profession. For doubtless it is true that, before the introduction of anæsthetics, patients sometimes died from syncope upon the operating table both immediately before and after the operation was commenced, and under conditions and circumstances which, in modern times, when anæsthetics are almost universally employed in operations, would be not unnaturally described and regarded as deaths from chloroform. Formerly, such sudden deaths under surgical operations, do not seem to have been looked upon as matters of moment, because, in fact, no special pathological or practical interest was attached to their occurence. They were simply regarded as inevitable accidents, and are usually only incidentally alluded to, when alluded to at all, by surgical authors, provided they illustrate some special observation or opinions on the part of the writer. Thus, as showing how "violence alone, without the loss of blood, may often produce immediate fatal effects," John Hunter makes the following observations on ten illustrative cases:

If, in the preceding cases, chloroform had happened to be employed, the fatal results would naturally, by most minds have been attributed to the anæsthetic, and not to the operation, or the condition of the mind or body connected with the operation. Such cases teach us at least, that caution is required in our reasoning and inferences, seeing that death may occur, and has occurred, in operations without chloroform, and with phenomena quite similar to those ascribed to the action of chloroform. Most of the stronger drugs in the *Pharmacopœia* as opium, elaterium, antimony, mercury, etc., are, proportionally to the number of cases in which they are used in full doses, as fatal as or more fatal than chloroform, but they are not so sudden, and hence not so infinitely appalling in their dangerous and fatal effects. The number, for example, of lives lost yearly by the poisonous effects of opium is much greater than that lost by chloroform.* At our different drug manufactories in Edinburg, we have upward of two million doses of chloroform manufactured annually, and much is also made elsewhere; yet how rarely does a fatal result follow its use! Is there any other common or potent drug which could be given in full doses in two millions of instances yearly, with greater impunity?—*British Med. Journal.—Med. Gazette.*

---

*In 1840, out of every 1,000,000 living in England and Wales, 24 were poisoned by opium and 22 by other medicines improperly given to children below the age of five years. (See *Seventh Annual Report of the Registrar-General*, p. 82) In England and Wales, in the five years from 1863 to 1867, there were poisoned by preparations of opium, 632 individuals; by salts of lead, 242; by overdoses of medicine, 52; by strychnine, 41; etc. There were drowned during the same period while bathing, 707 persons; while sliding or skating, 116; burnt to death by clothes taking fire, 2,194; killed by falls in walking 194; suffocated by bed clothes, 2,332 children; suffocated by overlaying, 682; died from navel hemorrhage, 572; etc. (See *Thirteenth Annual Report of the Registrar General.*)

